# Gush

**Published:** 1885-11

**Authors:** 


### GUSH.

### A REPORTER GOES THROUGH A DOCTOR'S OFFICE AND DESCRIBES THINGS.

A gushing reporter was shown through the newly fitted up offices of a firm of our esteemed subscribers and friends, and he gushed most energetically in a two column effusion. Amongst other things, on being shown a painting of Ambrose Pare applying the first ligature, he said, "Formerly the surgeons of the 14th century had most imperfect and crude ideas regarding the staunching of blood,"—from which we may infer that the surgeons of the 14 (?) century have now a better knowledge of such things. [Note—Pare was born 1615.—Ed.]

On viewing "Harvey demonstrating the circulation of the blood" this gem of a reporter of the purest stupidity serene, gets off the following, which, for gush, and bad use of the king's English, we will enter as the champion-of-the-world-for-the-belt specimen,—listen.

"We cannot, with such a name as Harvey before us, say less, than if a man die without making any impression on the age in which he lives [that's good by itself Ed.] perchance he may arouse the world by an original thought; such a man was Harvey." That is to say, Harvey didn't make any impression on the age in which he *lived* after he *died*, but after he died he aroused the world by an original thought.

But here is a creamy sentence ; listen to its silvery (or rather its milky) flow and ripple as it gushes forth like "a fountain i' the midst of roses."

"And one to look upon a copy illustrating the magnificent face of Harvey with the noble prince and the joker of that prince, King Charles the XII, would secure a bright page in the memory of those who have read the history of those times."

Ahem !—Language is powerless to do justice to the sublimity of the ridiculousness of that sentence. Let's see ;—for illustration; suppose *you* my reader, are a reader of "the history of those times" while we are the "one to look upon a copy illustrating the magnificent face with the n. p. and the j. of that p. King Charles XII. Why, *we*, by looking upon that copy, at once "secure a bright page" in *your* memory,—don't you see ?

But the following would shame A. Ward in his palmiest days; it approaches infinity, in the immensity of its whatness. This was our gem of a reporter's soliloquy in gazing at the pictures in the Doctor's office :

"Those who look upon a picture or an engraving [?] may not be impressed properly with the life that there is in it ; [so far, so good] but, to the literary and scientific man [like the reporter, for instance] he [?] will tell you there are lives that so much of brains, so much of work, so much of enthusiasm, that had they not done something to impress running literature, still did something to make an impress on the current literature of the age that stamps them a genius." [! ! !]

Now, that it is positively *soul stirring*, overwhelming, *ravishing* ! Oh, we wish we could *invent* a word that, as Sam Weller said, "means more" than those hackneyed phrases. Young men, and the unborn generations, should learn that "by heart," and treasure it up as a most precious precept, and strive by all means to leave an impress on the *current* literature, even if they fail to make a dent in the *running* literature.

The climax of absurdity was only reached by this gushing reporter, however, after having flattered the Doctors, told how much the instruments cost, and the furniture, and the amount of practice the Doctors do, and how they are overworked ; and described the library and the showcases of instruments ; and told about the "tumors and ovarian specimens" they had removed, and how much one weighed, etc., he said: "One cannot speak too highly of the large variety of surgical instruments owned by these Doctors. They perform four and five operations every day, and to insure perfect cleanliness the table is washed off with chloride of zinc every Saturday at three o'clock." [! ! !]

We cannot compare this fulsome fulmination of this bright reporter, who will have Harvey's home in the "Evon Isle," to aught else on earth, or in the heavens above, or the waters beneath, except to the grandiloquent, spontaneous, *ex tempore* overflowing of the newly arrived Frenchman's enthusiasm—who exclaimed, "Who but the g-r-e-eat Christopher Columbo could have discovered these mighty ocean? and planted the liberty of speech on thou brow, oh Columbia!"

It is to be regretted that these distinguished and most worthy physicians could not anticipate the appearance of this effusion, well meant probably, but damaging, or no doubt their well known good taste and modesty would have prompted them to suppress it. In that event though, we and our readers would have been deprived of a literary "jamboree," which we may say, for high-stilted *bosh*, beats the record.

